# Parameter Reduction and Optimisation for Point Cloud and Occupancy Mapping Algorithms

**DOI:** 10.3390/s21217004

**Published:** 2021-10-22

**Authors:** Yu Miao, Alan Hunter, Ioannis Georgilas

**Affiliations:** Department of Mechanical Engineering, University of Bath, Bath BA2 7AY, UK; A.J.Hunter@bath.ac.uk (A.H.); I.Georgilas@bath.ac.uk (I.G.)

**Keywords:** mapping, SLAM, data sets for SLAM

## Abstract

Occupancy mapping is widely used to generate volumetric 3D environment models from point clouds, informing a robotic platform which parts of the environment are free and which are not. The selection of the parameters that govern the point cloud generation algorithms and mapping algorithms affects the process and the quality of the final map. Although previous studies have been reported in the literature on optimising major parameter configurations, research in the process to identify optimal parameter sets to achieve best occupancy mapping performance remains limited. The current work aims to fill this gap with a two-step principled methodology that first identifies the most significant parameters by conducting Neighbourhood Component Analysis on all parameters and then optimise those using grid search with the area under the Receiver Operating Characteristic curve. This study is conducted on 20 data sets with specially designed targets, providing precise ground truths for evaluation purposes. The methodology is tested on OctoMap with point clouds created by applying StereoSGBM on the images from a stereo camera. A clear indication can be seen that mapping parameters are more important than point cloud generation parameters. Moreover, up to 15% improvement in mapping performance can be achieved over default parameters.

## 1. Introduction

In robotics, occupancy maps have a wide range of applications, including spatial representation of the real world [1], navigation [2], motion planning [3] and autonomous driving [4]. Maps are commonly generated from point clouds with a variety of sensors such as LIDAR [5], RGB-D cameras [6] and stereo cameras [7]. One popular occupancy mapping algorithm is OctoMap generating occupancy maps from these point clouds. OctoMap is an efficient and flexible framework for 3D environment mapping [8] and is using the octree structure [9] and its cubic nodes to provide a representation of the 3D space.

The operation of OctoMap is governed by several parameters, the choice of which will affect the performance of the mapping algorithm. The default parameters are introduced in [8]; however, there is no clear evidence to show they are the optimal ones. In that work, there is no systematic method provided to test these parameters and only clamping parameters are analysed by Kullback–Leibler divergence (KLD) in terms of map accuracy and compression. With a higher clamping threshold, a map can be further compressed but at the cost of losing map confidence.

In [8], point clouds are generated by a LIDAR and the algorithm accuracy is defined as the percentage of correctly mapped nodes in all 3D scans from the sensor. A node counts as correctly mapped if it has the same occupancy state in the evaluated scan and the pre-built map generated by all or part of the scans. Although this definition of accuracy can make comparison easy, it cannot illustrate how the model is right or wrong in absolute terms in a confusion matrix as per [10]. Moreover, in [8], the pre-built map and the maps to be evaluated are generated by the same data set. On the one hand, the measurements in the data set may contain noise and thus cannot represent the ground truth very well. On the other hand, the ground truth is better to be generated in a different way than with the data set itself. A better alternative would have been the use of a measured ground truth based on the measurements with a measuring device.

Another potential limitation in [8] is that point clouds may not be obtained directly from 3D sensors, e.g., LIDAR, but using cheaper solutions, for example, a stereo camera. In this case the point clouds need to be reconstructed following an algorithm. As a result, parameters for point cloud generation affect the quality of point clouds and thus have a potential impact on mapping performance.

Given the usefulness of occupancy maps, the wide adoption of OctoMap as a mapping algorithm, and the various sensor approaches to create point clouds, the impact of the different parameters needs to be evaluated and well understood. This work aims to achieve this by investigating the effect of those parameters in the performance of the mapping algorithm. The highlights of this paper are:

- A systematic method for parameter reduction and optimisation based on Neighbourhood Component Analysis (NCA) [11] and grid search;
- The use of a Receiver Operating Characteristic (ROC) curve variant as a performance metric to deal with skewed data in a confusion matrix due to mainly free space;
- A controlled procedure for data collection with two different environments and two different object textures to evaluate the effect of the scene to the process;
- Implementing pixel connectivity [12] in image processing for node classification to deal with point fluctuation;
- Using the StereoSGBM algorithm [13] on images derived by a stereo camera to demonstrate the effectiveness of the proposed approach, strengthening the potential of the methodology to be applicable with a variety of systems and sensors.

Initially we provide a detailed discussion of point cloud parameters and OctoMap parameters in Section 2. Our proposed method for parameter reduction based on NCA is presented in Section 3, followed by the grid search optimisation. A simple mapping approach as a proxy for the cleanness of point clouds is also introduced. The details of the 20 data sets collected in the outdoor environments with the controlled experimental procedure and measured ground truths are introduced in Section 4. The parameter reduction and optimisation results are presented in Section 4 as well. In Section 4.7, the key findings of the higher impact of mapping parameters compared to point cloud parameters, and the improvement by optimisation of up to 15% over the default OctoMap parameters are discussed.

## 2. Background

### 2.1. 3D Point Cloud Generation from a Stereo Camera

The first step in most mapping applications is to create point clouds from raw sensor data. For the case of a stereo camera the StereoSGBM algorithm [13] in the OpenCV library can generate the disparity map of left and right images. Then the point cloud can be reconstructed by the stereo camera model from this disparity map. The parameters in StereoSGBM algorithm in OpenCV are as follows.

- $d_{\min}$ is the minimum possible disparity value. It is normally set to 0 and should be adjusted accordingly when rectification algorithms can shift images.
- $d_{n}$ is the difference between maximum disparity and minimum disparity. It must be a number greater than 0 and divisible by 16.
- $B_{s}$ is the block size for matching two images. It must be an odd number no less than 1.
- $P_{1}$ is the first parameter for disparity smoothness control. A reasonably good sample in OpenCV is $P_{1}=8N_{c}B_{s}^{2}$, where $N_{c}$ is the number of image channels.
- $P_{2}$ is the second parameter for disparity smoothness control. A reasonably good sample in OpenCV is $P_{2}=32N_{c}B_{s}^{2}$.
- $d_{m}$ is the maximum allowed difference in the left-right disparity check. A non-positive value will disable the check.
- *C* is the truncation value for pre-filtered image pixels.
- $r_{u}$ is the margin in percentage.
- $d_{w}$ is the maximum size of smooth disparity regions to consider noise speckles and invalidate. 0 will disable speckle filtering.
- $d_{v}$ is the maximum variation in disparity. Normally, 1 or 2 for $d_{v}$ is good enough.
- mode is an option to run the full-scale two-pass dynamic programming algorithm, which is set to false by default.

### 2.2. OctoMap Algorithm

The method for occupancy map generation which we will be investigating in this work is OctoMap. The detailed description of the update policy of OctoMap can be found in [8], but here a brief analysis of the key steps for our investigation will be given.

OctoMap integrates sensor readings with occupancy grid mapping in a method introduced in [14]. The probability of a node can be modelled by:

(1) $$p\left(m_{i}|z_{1:t}\right)={\left[1+\frac{1-p(m_{i}|z_{t})}{p(m_{i}|z_{t})}\frac{1-p(m_{i}|z_{1:t-1})}{p(m_{i}|z_{1:t-1})}\frac{p(m_{i})}{1-p(m_{i})}\right]}^{-1}\;,$$

where $m_{i}$ is a node in the map and $z_{1:t}$ denotes a set of sensor measurements to time *t*. $p(m_{i}|z_{t})$ is the probability given current measurement $z_{t}$, and $p(m_{i})$ is the prior probability and equals to 0.5.

OctoMap simplifies (1) with the log-odds notation:

(2) $$l\left(x\right)=\ln\left(\frac{x}{1-x}\right)\;.$$

Then the probability of a node given sensor measurements $z_{1:t}$ can be denoted as:

(3) $$l\left(m_{i}|z_{1:t}\right)=l\left(m_{i}|z_{1:t-1}\right)+l\left(m_{i}|z_{t}\right)\;,$$

where $l(m_{i}|z_{t})$ is the inverse sensor model. A ray-cast operation will be performed from the sensor origin to the endpoints to determine which nodes should be updated. The inverse sensor model in OctoMap is defined as:

(4) $$l\left(m_{i}|z_{t}\right)=\{\begin{array}{cc} l_{occ} & \mathrm{if}\;\mathrm{beam}\;\mathrm{is}\;\mathrm{reflected}\;\mathrm{within}\;\mathrm{volume}\\ l_{free} & \mathrm{if}\;\mathrm{beam}\;\mathrm{traversed}\;\mathrm{volume} \end{array}\;,$$

where $l_{occ}$ and $l_{free}$ are the log-odds values to update occupied and free cells, respectively. A clamping update policy proposed in [15] is applied in OctoMap to set limitations on the log-odds value:

(5) $$l\left(m_{i}|z_{t}\right)=\max\left(\min\left(l\left(m_{i}|z_{1:t-1}\right)+l\left(m_{i}|z_{t}\right),l_{\max}\right),l_{\min}\right)\;,$$

where $l_{\max}$ and $l_{\min}$ are lower and upper bounds in log-odds, respectively.

Based on the above OctoMap algorithm, the parameters which have an impact on the occupancy probability of a node are listed as follows.

- $p_{\max}$ is the upper clamping threshold. It is the upper bound of the occupancy probability.
- $p_{h}$ is the probability for a “hit”. If a node contains endpoints, a “hit” will be integrated to the node.
- $p_{t}$ is the occupancy threshold. The node will be marked as occupied if its occupancy probability is greater than the threshold.
- $p_{m}$ is the probability for a “miss”. If a node is traversed by rays, a “miss" will be integrated to the node.
- $p_{\min}$ is the lower clamping threshold. It is the lower bound on the occupancy probability.

## 3. Method

In this work, we are proposing a systematic method to evaluate and identify the optimum set of parameters for occupancy mapping. This is achieved by the reduction of parameter space using NCA and the optimisation of Octomap parameters with a grid search of the parameter space. The respective steps are presented in this section.

### 3.1. Parameter Space Considerations

The parameter space for the analysis will be generated in the following way. Each parameter will be generated by three values, i.e., minimum, maximum and step, as well as the algorithm-required relations with other parameters. The possible values of a parameter *T* can be denoted as:

(6) TTmax−TminTs| T=Tmin+(i−1)Ts, i<Tmax−TminTs+1, i∈ℕ+ ,

where $T_{\max}$ and $T_{\min}$ are upper and lower bounds on the parameter, and $T_{s}$ is the step.

Two functions are defined to describe the combinations of parameters. One function is:

(7) $$f\left(T\right)=\left\lfloor \frac{\max(T)-\min(T)}{T_{s}}\right\rfloor +1\;.$$

The other one is:

(8) g(T,T′,n)=minmin(T)+(n−1)Ts−min(T′)Ts′, f(T′)−1min(T)+(n−1)Ts−min(T′)Ts′+1 ,

where *T* and $T^{\prime }$ are respective collections of possible values for two parameters, $T_{s}$ and $T_{s}^{\prime }$ are corresponding steps, and *n* is an integer.

With the above definitions, we can easily get the number of all the combinations of point cloud parameters as:

(9) $$N_{p}=f\left(d_{\min}\right)f\left(d_{n}\right)f\left(B_{s}\right)f\left(P_{1}\right)f\left(P_{2}\right)f\left(d_{m}\right)f\left(C\right)f\left(r_{u}\right)f\left(d_{w}\right)f\left(d_{v}\right)f\left(mode\right)\;.$$

In addition to the above grid of parameters, by considering upper and lower bounds on the probability, a reasonable set of OctoMap parameters should satisfy:

(10) $$\left\{ \begin{array}{c} 1>p_{\max}\geq p_{h}\geq 0.5>p_{m}\geq p_{\min}>0\\ p_{\max}\geq p_{t}\geq p_{\min} \end{array}\;.\right.$$

Here we avoid $p_{\max}=1$ and $p_{\min}=0$ in case the log-odds probability is not a number. From the above relationship, we can infer that:

(11) $$\left\{ \begin{array}{c} \min\left(p_{\max}\right)\geq \min\left(p_{h}\right)>\min\left(p_{m}\right)\geq \min\left(p_{\min}\right)\\ \min\left(p_{\max}\right)\geq \min\left(p_{t}\right)\geq \min\left(p_{\min}\right) \end{array}\;.\right.$$

Then the number of combinations of OctoMap parameters is:

(12) $$N_{o}=\sum_{i=1}^{f(p_{\max})}\sum_{j=1}^{f(p_{m})}\sum_{k=1}^{g(p_{m},p_{\min},j)}g\left(p_{\max},p_{h},i\right)f\left(p_{t}\right)\;,$$

where the possible values of $p_{t}$ correspond to $p_{\max}$ value: $\min\left(p_{\max}\right)+\left(i-1\right)\tau \left(p_{\max}\right)$ and $p_{\min}$ value: $\min\left(p_{\min}\right)+\left(k-1\right)\tau \left(p_{\min}\right)$, and $\tau \left(T\right)=T_{s}$.

Parameters in the StereoSGBM algorithm and OctoMap will be combined and tested on the different data sets, the details of which will be introduced in Section 4. Let $N_{d}$ denote the number of data sets, a random permutation of the indices of all the combinations will be generated and divided into $N_{d}$ groups. The number of combinations for each data set is:

(13) $$N_{t}=\frac{N_{p}N_{o}}{N_{d}}\;.$$

### 3.2. Parameter Reduction Using NCA

OctoMap can be treated as a binary classifier since it classifies nodes into occupied and free ones. In machine learning, a confusion matrix, also called a contingency table, is a table layout to describe the performance of a classification algorithm [10,16]. A confusion matrix summarises the number of true positives (TP), false positives (FP), true negatives (TN) and false negatives (FN). Created by plotting the true positive rate (TPR) against the false positive rate (FPR), an ROC curve summarises a confusion matrix at different thresholds and is commonly used to demonstrate the diagnostic ability of a binary classifier [17]. The area under the curve (AUC) derived by an ROC curve is used as a performance measure.

However, the traditional AUC-ROC method is not effective in dealing with unbalanced data sets in which elements in one class are more than in others. For example, in empty scenes, most elements in a map are classified as TNs, which will distort the ROC curve since only a small portion of the curve is relevant to the real test. In [18], ROC surface (ROCS) is proposed to address this issue. Three metrics, i.e., TPR, FPR and the true discovery rate (TDR), are used to generate a three-dimensional surface by projecting FPR-TPR-TDR curve to TPR-TDR plane. In the ROCS method, AUC is replaced by the volume under the surface (VUS). However, the bottom surface is effectively defined by the traditional ROC curve. When an unbalanced data set is heavily skewed by TNs, with a reasonable mapping result, the AUC derived by the curve in the bottom surface is approximately equal to 1 since the FPR of most points on the curve is approximately 1 and varies in a very small range. In addition, TDR can be derived by false discovery rate (FDR). Therefore, FPR can be ignored and TDR can be replaced with FDR to reduce the metrics from three dimensions to two dimensions. In this work, we use the metrics in the ROC variant, the TPR-FDR curve as in [19,20], to evaluate the quality of a map.

For the occupancy map derived by each combination of parameters, we compute the corresponding TPR and FDR by the number of nodes in the four categories. NCA feature selection [11] will be applied to compute the weight of each parameter in the two metrics. Weights are normalised as in [11] to compare the results derived by different data sets.

### 3.3. OctoMap Parameter Optimisation

To evaluate the mapping approach performance, we need a consistent evaluation of the quality of the point cloud used. Given different combinations of point cloud parameters will give different quality of point clouds, a naive mapping policy is implemented here as a proxy to point cloud quality.

A node will be marked as occupied if it contains points, while a node will be marked as free if it contains no points and is traversed by rays cast from the sensor to end points. Once a node is marked as occupied, it cannot be converted to a free node. This is a simple and naive way to generate an occupancy map, which is not affected by parameters. In this update policy, the states of all potentially occupied nodes are guaranteed to be occupied. As a result, the FDR of the generated map is the proxy for the cleanness of the point cloud set. The higher the FDR is, the more nodes have been incorrectly identified as occupied due to noise points being in empty space.

We rank point cloud sets produced by different parameters from each data set by the FDR derived by the naive mapping approach and then select five of those point cloud sets. Then a series of combinations of OctoMap parameters will be generated and implemented on the selected point cloud sets. We use the AUC of TPR-FDR variant to optimise parameters. The optimal AUC will be compared with that derived by default parameters as suggested in [8]. In this work, data sets introduced in Section 4 will be randomly divided into two groups for training and test purposes. The optimal parameter set derived by training will be validated on test data sets.

## 4. Experiments

### 4.1. Test Scenes and Targets

In real-world applications, environments and objects can be of rich features or lack of features. The experiments in this work are conducted in two different environments, in front of buildings and in a parking lot. Boxes with different textures on the external surfaces are the targets to be explored. The boxes have a plain cardboard texture or are covered with Voronoi diagrams to allow the investigation of the impact of textures. The above settings are used to simulate different conditions in the real world. We aim to provide small test scenes to mediate between mapping algorithms and real applications where the scene to be explored is normally large, so that parameters can be tuned before being applied.

The Voronoi diagram is generated by dividing a plane into regions where all the points in each region is closer to one point in the given point set to any other point in the set [21]. To cover the external surfaces of the boxes, Voronoi diagrams are printed on A0 posters with 300 DPI and then cropped to match the size of the boxes. The average size of the polygons in the diagram is about 3 × 3 cm. Each polygon is filled with a random colour. Since patterns are randomly generated, the diagram is different in each poster.

**Figure 1:**
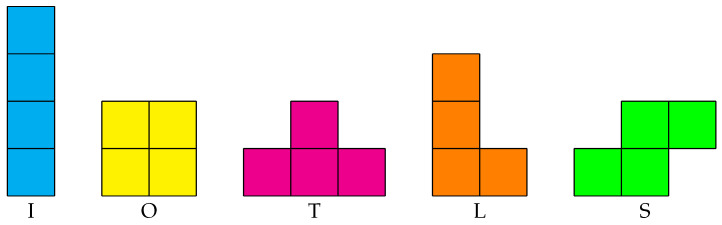
Free tetrominoes.

Inspired by the Tetris game, we can create several different layouts with a pair of boxes. There are seven one-sided tetrominoes in Teris game, including two enantiomeric pairs [22]. These shapes are not superimposable in 2D space and can be translated, rotated but not reflected. By excluding one of the shapes from each enantiomeric pair, we can get five free tetrominoes [23]. As shown in Figure 1, the shapes of five free tetrominoes are I, O, T, L and S. Based on these free tetrominoes, we can create five layouts with two boxes.

### 4.2. Data Collection

**Figure 2:**
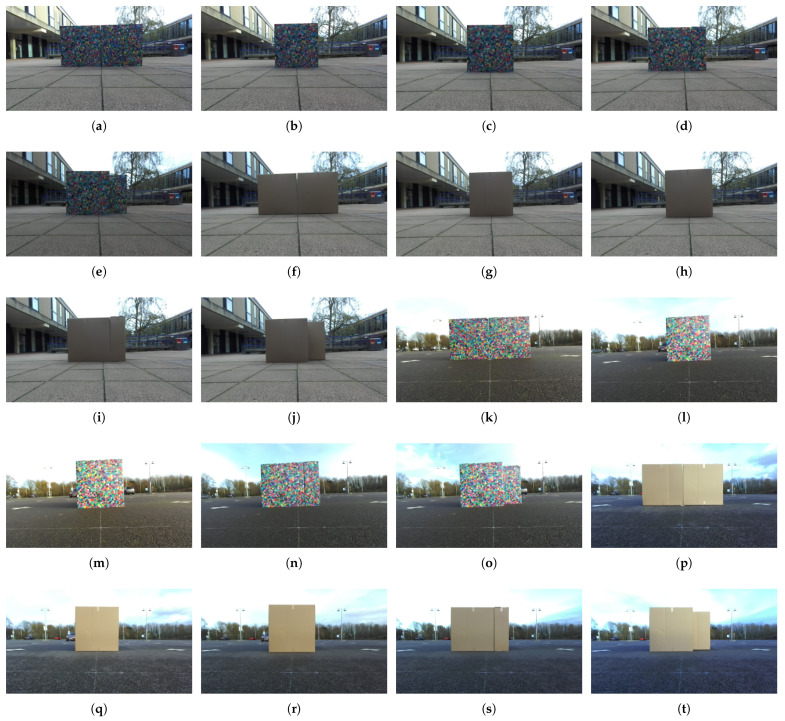
Boxes of five layouts and two textures in two environments. (**a**–**e**) I, O, T, L and S layout boxes with Voronoi diagrams in front of buildings. (**f**–**j**) I, O, T, L and S layout plain boxes in front of buildings. (**k**–**o**) I, O, T, L and S layout boxes with Voronoi diagrams in the parking lot. (**p**–**t**) I, O, T, L and S layout plain boxes in the parking lot.

**Figure 3:**
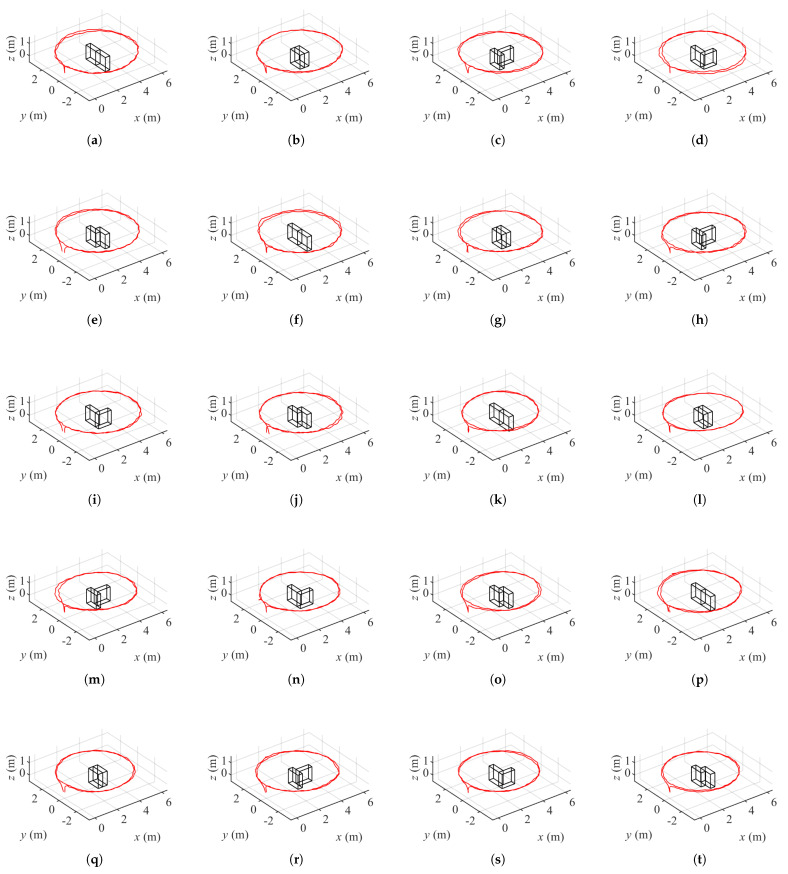
Camera trajectories of boxes of five layouts and two textures in two environments. (**a**–**e**) I, O, T, L and S layout boxes with Voronoi diagrams in front of buildings. (**f**–**j**) I, O, T, L and S layout plain boxes in front of buildings. (**k**–**o**) I, O, T, L and S layout boxes with Voronoi diagrams in the parking lot. (**p**–**t**) I, O, T, L and S layout plain boxes in the parking lot.

Considering two environments, two textures and five layouts, 20 data sets will be collected with a controlled procedure. A circle of approximate radius 2.96 m is drawn on the ground. Then the boxes are put in the centre of the circle and arranged in a layout as one of the free tetrominoes. The centre of the circle is also the centroid of the bottom surface of each layout. A ZED stereo camera (Stereo Labs, San Francisco, CA, USA) is placed in front of the boxes and the initial relative position between the camera and boxes is measured. Then the camera moves along the circle orbiting around the objects twice to record videos at HD resolution (2560 × 720 pixels for a stereo camera). Figure 2 shows boxes of different layouts and textures in two environments, and corresponding camera trajectories are presented in Figure 3. The origin point in each coordinate system is the initial position of the camera. The camera moves in a anti-clock direction.

### 4.3. Parameter Space for Analysis

In Table 1 and Table 2, the parameter space minimum, maximum and step are given for point cloud generation, and OctoMap parameter reduction and optimisation.

The configuration of point cloud parameters is shown in Table 1. The steps for the parameters whose values are constant or determined by other parameters are not required. Specifically, $d_{\min}$, $d_{n}$ and mode are constants. $d_{\min}=0$ since the camera is well calibrated at the factory. $d_{n}$ controls visible depth and has no impacts on the quality of the disparity map and is set to constant to make point clouds comparable when the other parameters are varied. mode is set as true to improve the quality of disparity maps. $P_{1}$ and $P_{2}$ are determined by image channel number $N_{c}$ and parameter $B_{s}$. $d_{w}$ is up to 1000 following recommended setting for relatively large object targets. As a result of those minimum, maximum and steps the number of combinations of point cloud parameters is 1600.

Table 2 shows the configuration for the OctoMap parameter space. For parameter $p_{t}$ from $p_{\min}$ to $p_{\max}$ we are investigating two steps, a step of 0.12 for the reduction analysis and a step of $(p_{\max}-p_{\min})/8$ for optimisation, with 9 points for generating the ROC curve. If the metric of a point is not a number, we exclude that point. The step size of 0.12 and 9 points were decided following a limited testing of the cases using step size 0.06 and 17 points. No obvious difference was found in results, but there was a significant computational time penalty. As a result, for the experiments reported here, the steps given in Table 2 will be used with OctoMap parameter combinations for reduction and optimisation being 1000 and 1350, respectively.

### 4.4. Experimental Method

**Figure 4:**
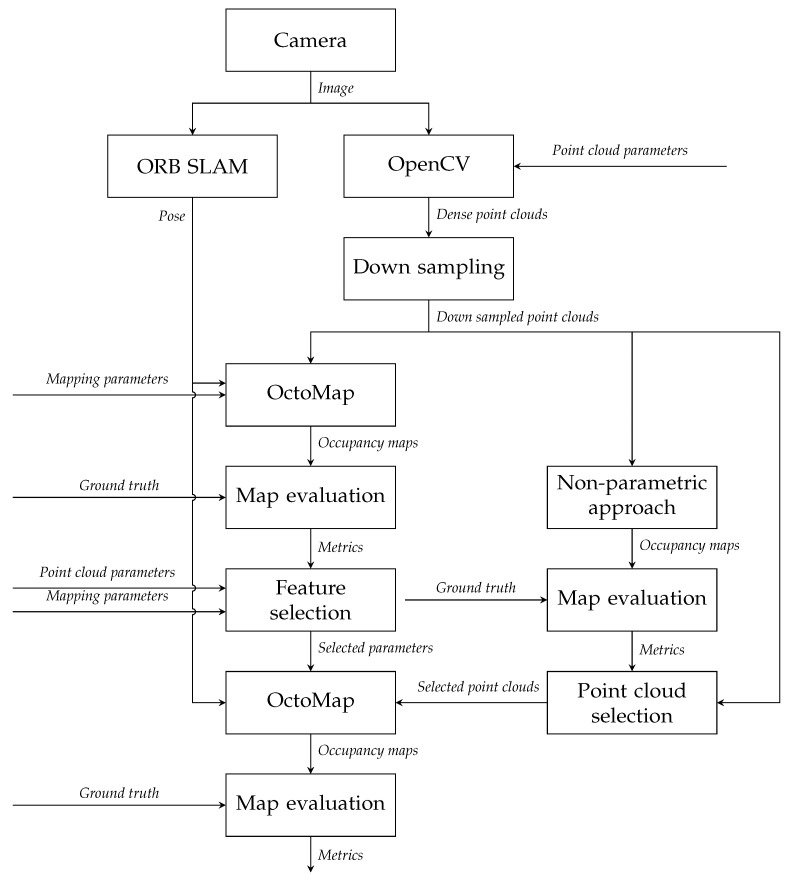
Design of experiments.

Parameter weights will be analysed to reduce the parameters of lower impacts and the residual most important parameters will be optimised to improve the mapping performance. An exhaustive test on point cloud and OctoMap parameters will be first performed to study the impact of each parameter. In our experiments, ORB-SLAM [24] is implemented to generate the poses of the camera. ORB-SLAM is considered to be the most complete feature-based monocular visual SLAM system and has been extended to stereo visual SLAM [24,25]. In this work, ORB parameters are of default values since the poses derived by ORB-SLAM are optimised when the loop is detected, and the parameters are for ORB feature extraction and out of the scope, and ORB parameters have been discussed in [26]. Figure 4 shows the design for experiments.

To generate an occupancy map, point clouds and corresponding poses are required. A series of keyframes is produced by ORB-SLAM and with the time stamps of those keyframes the images can be matched with their respective keyframe poses. The StereoSGBM algorithm is implemented on the images of keyframes to produce disparity maps, from which point clouds can be reconstructed. Points will be preserved if their distances are within 8 m from the camera in the principal axis.

Point clouds are down sampled by the Voxel filter in Point Cloud Library (PCL) [27] at resolution 0.1 m before they are processed by OctoMap, to reduce computational time. The leaf size in OctoMap is also set to 0.1 m. The resolution is chosen based on the scale of test scenes and the size of targets. If the leaf size is too big, the details on the external surface of an object will be lost. We also consider the fluctuation of the points. Since point clouds derived by stereo images are not perfect, points will fluctuate near their real positions. This leaf size can tolerate the fluctuation. Moreover, the resolution is constant here since maps of different leaf sizes are not comparable with the total number of nodes being different, even derived by the same point cloud set. Maximum range for how long individual beams are inserted is set to 4 m since the fluctuation of points is not serious within this range.

Occupancy maps derived by different combinations of parameters will be compared with ground truths to classify the nodes into TPs, FPs, TNs and FNs using the method introduced in Section 4.5. The performance metrics derived by the number of the nodes in each category along with different parameter sets will be analysed by NCA feature selection to determine which parameters should be neglected or optimised. The most important ones will be optimised on point cloud sets selected by the non-parametric naive approach explained in Section 3.3.

### 4.5. Node Classification

As shown in Figure 4, an occupancy map will be evaluated to compute performance metrics. A ground truth is needed as a reference to classify nodes in a map into the four categories. To generate the ground truth, we first measure the dimensions and locations of the targets in the real environment. With these measurements, we can generate point clouds containing points on the external surfaces of objects to produce an occupancy map as the ground truth. The nodes containing the external surfaces in the ground truth are marked as occupied while free nodes are marked accordingly. Occupied nodes normally form a shell and the space inside the shell is marked as unknown since the inside is not observable. If the quality of the data collected by a sensor is good enough, the corresponding occupancy map derived by a mapping algorithm should match well with this ground truth.

In our experiments, points may appear inside objects due to fluctuation. If the state of a node in a map is known but the corresponding node in the ground truth is unknown, we will ignore this node in the classification procedure. For a free node in an map, we will query the ground truth with the coordinates of the node centre. If the corresponding node in the ground truth is free, the node in the map will be marked as TN. Otherwise, it will be marked as FN. To deal with the fluctuation of points, the concept of pixel connectivity [12] in image processing is introduced to the node classification procedure. 26-connected pixels will be used to identify TP and FP instances. For an occupied node in the occupancy map, we can get the coordinates of the node centre and its 26-connected nodes. The ground truth will be queried by the node centre coordinates first and then the coordinates of the neighbourhood nodes. If the corresponding node in the ground truth is occupied, the occupied node in the generated map is successfully associated with this node and the query process stops. The node in the map will be marked as TP, while the node in the ground truth will be marked as associated and it cannot be associated with other occupied nodes in the generated map. On the contrary, an occupied node in the generated map will be marked as FP if the ground truth has been queried by corresponding 27 coordinates but none of the nodes can be associated.

### 4.6. Results

**Figure 5:**
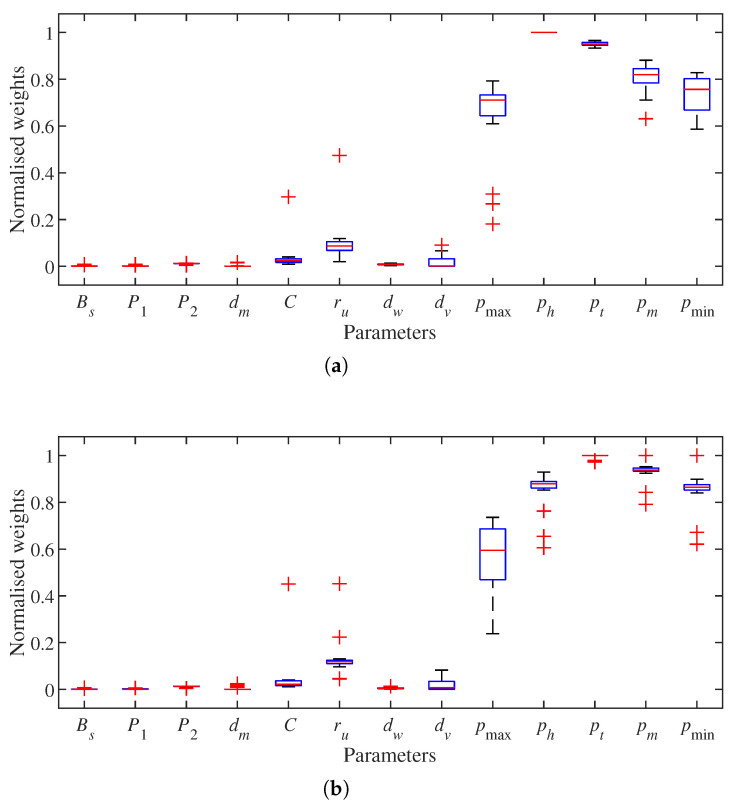
Normalised parameter weights for performance metrics true positive rate (TPR) and false discovery rate (FDR). (**a**) TPR. (**b**) FDR.

The first set of results is the weight of each of the point cloud and OctoMap parameters on the performance metrics TPR and FDR. Parameters of constant values are excluded from this analysis. With the configurations in Table 1 and Table 2, there are 80,000 combinations of point cloud parameters and OctoMap parameters for each data set. Parameter weights are calculated by implementing the NCA method on node classification results derived by the 20 data sets. Figure 5 shows the normalised weights of different parameters. The last five ones are OctoMap parameters, and they show higher weights under both performance metrics. For TPR, the majority of OctoMap parameter weights are over 0.6 while most point cloud parameter weights are under 0.2. FDR is similar to the case TPR with the weights of OctoMap parameters and point cloud parameters mostly being above 0.5 and under 0.2, respectively.

**Figure 6:**
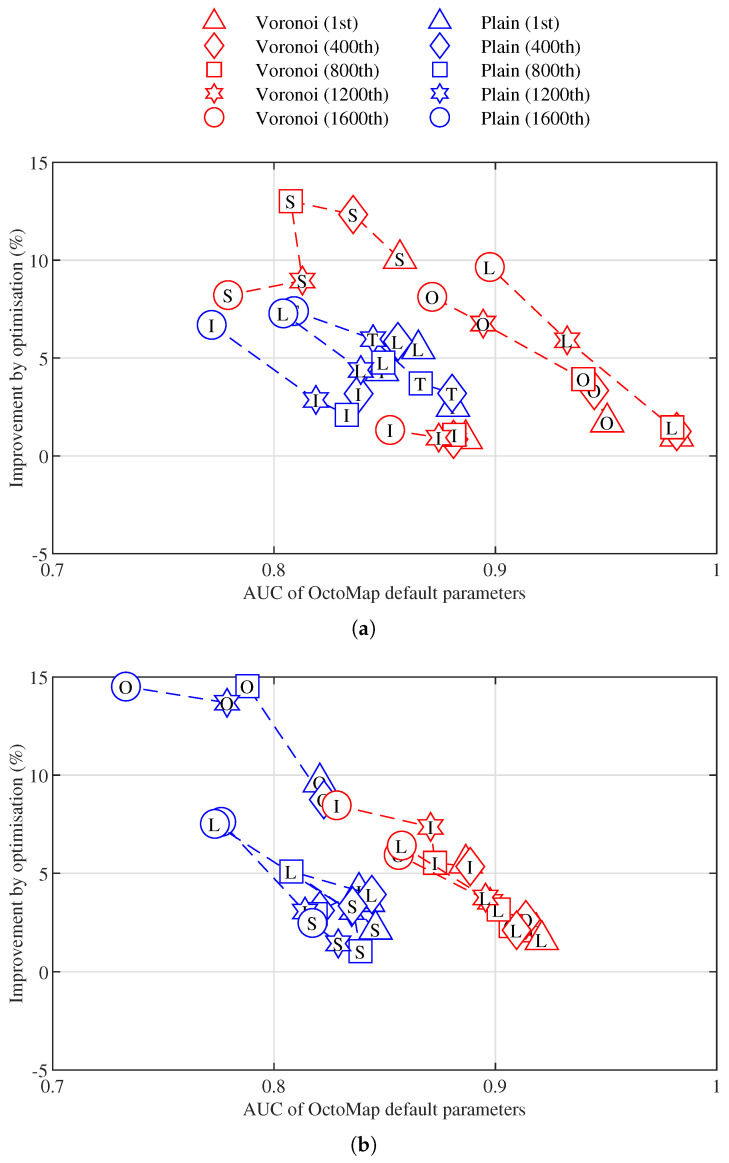
Improvement by optimisation over the area under the curve (AUC) of OctoMap default parameters on training data sets. (**a**) Building. (**b**) Parking lot.

Then we split 20 data sets into training and test groups, with 70% randomly selected for optimisation and the other 30% for validation. Since OctoMap parameters have higher weights, we only perform optimisation on these parameters with the training data sets. For each data set, 1600 sets of point clouds can be generated with the parameter configuration in Table 1 and ranked by the simple non-parametric mapping approach from Section 3.3. We choose the 1st, 400th, 800th, 1200th and 1600th (a lower number indicates better quality, i.e., cleaner point clouds) ranked point cloud sets from each data set to perform the optimisation of OctoMap parameters, of which the grid parameter space is generated by Table 2 and the relations in Section 3.1. The AUC of TPR-FDR curve specified in Section 3.3 is used as the performance measure for optimisation. The results of optimisation against OctoMap default parameters in [8] on the training data sets are presented in Figure 6. By optimisation, we can gain improvements on all cases in the two different environments, with highest improvement over default parameters of up to 15%. Overall, the AUC derived by default parameters using the building data set (Figure 6a) is better than that using the parking lot data set (Figure 6b).

**Figure 7:**
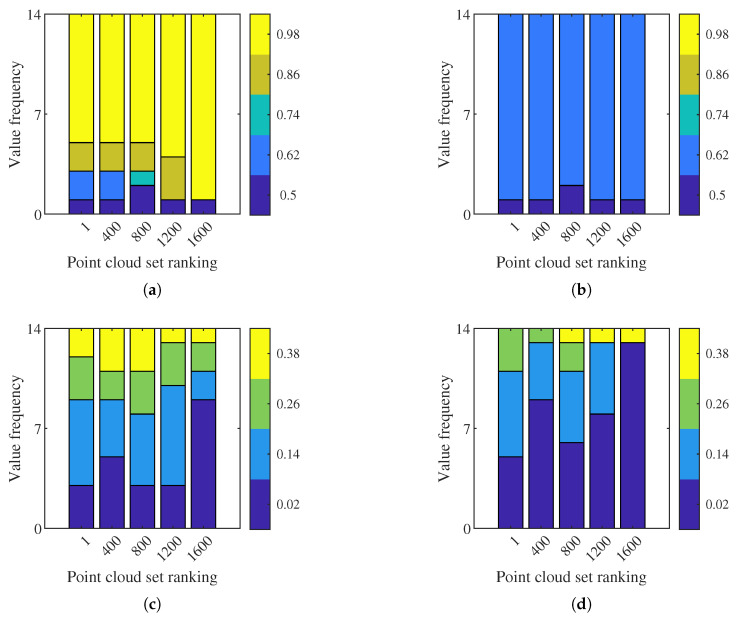
Frequency of optimal values of OctoMap parameters against point cloud set ranking on training data sets. (**a**) $p_{\max}$. (**b**) $p_{h}$. (**c**) $p_{m}$. (**d**) $p_{\min}$.

We also analyse the frequency of the optimal values of OctoMap parameters in the optimisation results. The optimal parameters are divided into five groups according to the point cloud set ranking. The frequency of the optimal values of each parameter is presented in Figure 7. There is not a obvious change in $p_{\max}$ and $p_{h}$ with the ranking (quality) of point cloud set. Overall, these two parameters are dominated by 0.98 and 0.62, respectively. For $p_{m}$ and $p_{\min}$, the optimal parameters tend to be smaller as the quality of point clouds becomes worse. Frequency of smaller values is higher than larger ones, especially when the point cloud quality degrades.

**Figure 8:**
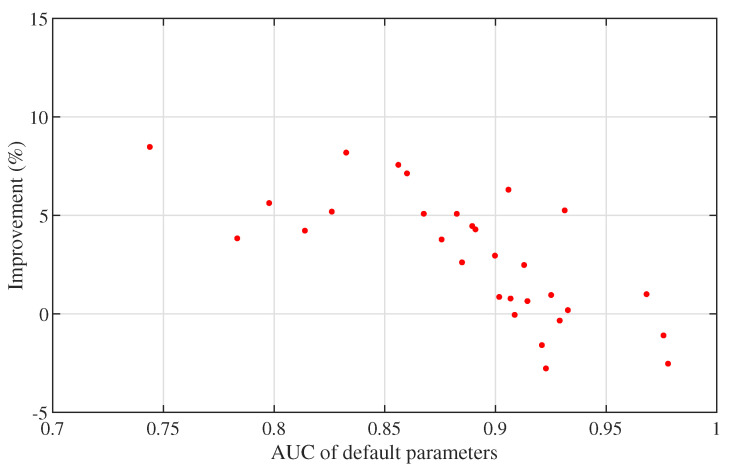
Cross-validation on test data sets.

Finally, we cross-validate the optimised results using the test data sets and the findings from the parameter frequency analysis in Figure 7. Figure 8 shows the validation on testing data sets with the most frequent values of $p_{\max}$, $p_{h}$, $p_{m}$ and $p_{\min}$ derived by training at 0.98, 0.62, 0.14 and 0.02, respectively. The improvement increases to 9% as AUC derived by default parameters decreases, but can be negative when AUC is already relatively large.

### 4.7. Discussion

OctoMap parameters have a higher impact on mapping performance than point cloud parameters. The optimisation on OctoMap parameters shows an improvement of up to 15% over default parameters. Overall, the improvement increases when the AUC of default parameters decreases. The mapping performance in the environment with buildings is better than that in the parking lot since there are more objects to provide image features. The performance also benefits from the rich features introduced by Voronoi diagrams. The baseline AUC generated by OctoMap default parameters in two environments is normally better when boxes are covered with Voronoi diagrams. There is not an obvious trend in the five tetromino layouts of boxes. However, a higher improvement can normally be achieved when the quality of the point clouds degrades in each data set.

The optimal values of $p_{\max}$ and $p_{\min}$ are similar for different point cloud sets, which reinforces our finding that parameters for point cloud generation are less important. Despite of the high consistency in these two parameters, one parameter set cannot achieve optimal improvement in all cases. With a higher baseline AUC, more occupied nodes can be preserved with bigger $p_{m}$ and $p_{\min}$. These occupied nodes mostly belong to TP category since point clouds corresponding to higher baseline AUC are usually of less noise, and thus the AUC can be improved. Loss in improvement can be observed when the values of $p_{m}$ and $p_{\min}$ increase since FPs are likely to be introduced, resulting in a worse FDR. On the contrary, smaller $p_{m}$ and $p_{\min}$ benefit mapping performance when point clouds are of low quality. On the one hand, the probability of a node drops faster with a smaller $p_{m}$. On the other hand, the probability can decrease further with a lower $p_{\min}$. Therefore, it would be more difficult for the probability to go beyond the threshold, benefiting FDR. Although point cloud parameters are of lower impacts, they still affect the mapping performance since $p_{m}$ and $p_{\min}$ do not show high consistency as $p_{\max}$ and $p_{h}$ when the point cloud quality degrades, especially when the rank of the point cloud set is 1600th. With worse point cloud quality, the impacts of the point cloud parameters might be overlooked when the performance is optimised by mapping parameters only.

## 5. Conclusions

In this paper, we present a framework of parameter reduction and optimisation for point cloud generation and occupancy mapping algorithms. Through NCA, the number of parameters can be reduced and the residual most important parameters can be optimised by investigating a grid parameter space. The proposed method is verified by the implementation of the StereoSGBM algorithm in OpenCV and OctoMap, and can be potentially extended to other systems. Results show that our approach is an effective in reducing parameters and robust in improving mapping performance. Our key findings are:

- Compared with point cloud parameters, mapping parameters have a higher impact on performance metrics TPR and FDR.
- Through grid search optimisation, the performance of OctoMap can be improved over default parameters.

In the future, we can consider optimising computation time. One limitation of our approach is that when the step is decreased, the number of combinations of parameters will increase dramatically. However, the optimisation results of our experiments in this paper do not benefit from decreasing the step. We will also test the proposed methodology on point clouds generated with other methods. In this work, results are presented based on the data sets collected in two outdoor environments. Different environments can be used to verify the effectiveness of the method in future work.

## Figures and Tables

**Table 1:** Configuration of point cloud parameters.

Parameter	Minimum	Maximum	Step
$d_{\min}$	0	0	N/A
$d_{n}$	80	80	N/A
$B_{s}$	3	15	4
$P_{1}$	$8N_{c}B_{s}^{2}$	$8N_{c}B_{s}^{2}$	N/A
$P_{2}$	$32N_{c}B_{s}^{2}$	$32N_{c}B_{s}^{2}$	N/A
$d_{m}$	0	1	1
*C*	10	50	10
$r_{u}$	5	35	10
$d_{w}$	200	1000	200
$d_{v}$	1	2	1
mode	true	true	N/A

**Table 2:** Configuration of OctoMap parameters.

Parameter	Minimum	Maximum	Step
$p_{\max}$	0.5	0.98	0.12
$p_{h}$	0.5	0.98	0.12
$p_{m}$	0.02	0.38	0.12
$p_{\min}$	0.02	0.38	0.12
$p_{t}$ ^a^	$p_{\min}$	$p_{\max}$	0.12
$p_{t}$ ^b^	$p_{\min}$	$p_{\max}$	$(p_{\max}-p_{\min})/8$

^a^ Configuration for parameter reduction. ^b^ Configuration for parameter optimisation.

## Data Availability

Data sets are available at https://doi.org/10.15125/BATH-00594, accessed on 10 September 2021 under the Creative Commons Attribution 4.0 license.
